# Retrospective study of hospitalized breast cancer patients in a Zhuhai-based hospital: analysis spanning over 20 years

**DOI:** 10.3389/fonc.2026.1706595

**Published:** 2026-03-25

**Authors:** Ruixin Fan, Yu Zheng, Xiangfeng Zhao, Juan Lin, Xueping Lu, Pan Hong, Weirong Chen

**Affiliations:** 1 Breast Surgery Department, Zhuhai Center for Maternal and Child Health Care (Zhuhai Women and Children’s Hospital), Zhuhai, Guangdong, China; 2 Department of Breast and Thyroid Surgery, Union Hospital, Tongji Medical College, Huazhong University of Science and Technology, Wuhan, China; 3 Department of Orthopaedic Surgery, Union Hospital, Tongji Medical College, Huazhong University of Science and Technology, Wuhan, China

**Keywords:** breast cancer, epidemiology, healthcare costs, retrospective study, treatment patterns

## Abstract

**Background:**

Breast cancer represents a significant global health burden with distinct epidemiological characteristics in China. This study analyzes the management evolution of breast cancer at a regional medical center in Southern China.

**Methods:**

A retrospective analysis was conducted of 5,052 breast cancer patients hospitalized from 2004 to 2024. Data encompassed demographics, clinical characteristics, treatment patterns, costs, and outcomes.

**Results:**

The cohort was predominantly female (99.5%) with a mean age of 50.5 years. Most cases (61.3%) were early-stage (Stage I), and 70.3% were covered by rural resident basic medical insurance. A 406% surge in hospitalizations occurred in 2022, while median length of stay significantly decreased from 16.9 to 4.8 days (p < 0.001). Treatment trends showed substantial increases in breast-conserving surgery (21.7% in 2024) and neoadjuvant chemotherapy. Total costs peaked in 2019 (¥31,201) then decreased by 58.9%, with out-of-pocket expenses declining from 49.2% to 37.4%. Logistic regression identified adjuvant chemotherapy as a strong positive predictor of cure (OR = 5.87, 95% CI: 3.14-10.99, p < 0.001).

**Conclusion:**

Findings confirm established patterns in age and tumor distribution while revealing regional variations in pathological profiles and surgical management.

## 1 Introduction

Breast cancer imposes a substantial global health burden. According to the latest global cancer data released by IARC, there were 2.261 million new cases of breast cancer worldwide in 2020, accounting for 11.7% of all new cancer cases and 24.5% of all new cancer cases in women. During the same period, there were 685,000 deaths from breast cancer among women, making it the leading cause of cancer incidence and mortality in women worldwide and the most common cancer endangering the health and lives of women globally (1).

Although the incidence rate of breast cancer in China is relatively low, the crude incidence rate has shown a year-by-year increase, making it one of the most significant cancers threatening the health and lives of Chinese women (2, 3). In China, breast cancer incidence rates have witnessed a rapid and steady increase in the past two decades, particularly in urbanized coastal areas (4–6). Guangdong Province exemplifies this trend, reporting the highest provincial incidence rate of 40.99 per 100,000 and a mortality rate of 6.95 per 100,000, making breast cancer the most common cancer among women in the area (7, 8). Located within the Pearl River Delta, Zhuhai represents a characteristic urban environment marked by rapid economic development, an aging population, and substantial domestic migration (9, 10).

Most existing studies have focused on major metropolitan areas such as Beijing, Shanghai, or Guangzhou, potentially overlooking regional variations in disease presentation and management. This 20-year retrospective study (2004-2024) aims to address this gap by examining the clinical characteristics, treatment patterns, and temporal trends among breast cancer patients hospitalized at Zhuhai Maternal and Child Health Hospital. Through detailed analysis of demographic profiles, diagnostic features, therapeutic interventions, and hospitalization patterns, this research seeks to provide valuable insights that can inform region-specific clinical strategies and public health initiatives, ultimately contributing to improved breast cancer care in southern China.

## 2 Methods

### 2.1 Study design and data source

This single-center retrospective study analyzed data from all breast surgery inpatients at Maternal and Child Health Hospital of Zhuhai, China, between 2004 and 2024. Each patient was included only once in the analysis, based on the first hospitalization for breast cancer during the study period; repeat hospitalizations were excluded. The study was approved by Ethics Committee of Zhuhai Maternal and Child Health Hospital (see **Appendix**). Due to the retrospective nature of the research, the requirement for informed consent was waived. All procedures involving human participants complied with the ethical standards of the Hospital’s Ethics Committee and the Declaration of Helsinki.

Demographic characteristics (like age, geographic origin, and payment methods) and clinical characteristics (like clinical diagnosis, tumor stage, and comorbidities) were analyzed. The study further examined trends in breast cancer hospitalization over the 20-year period in Zhuhai, focusing on treatment patterns, medical costs, and factors influencing therapeutic efficacy.

### 2.2 Data processing

All statistical analyses were performed using R version 4.2.3. Base R functions were used for data aggregation, sorting, and computation of maximum, minimum, mean, and standard deviation values.

Binary logistic regression was performed to evaluate factors associated with in-hospital treatment outcome. The outcome variable was derived from discharge status recorded in the hospital administrative system, where clinicians routinely categorize patients as “cured,” “improved,” “not improved,” or “death” based on clinical assessment at discharge. For modeling purposes, outcomes were dichotomized into favorable (cured/improved) and unfavorable (not improved/death). Although long-term endpoints such as survival and recurrence are emphasized in breast cancer guidelines, discharge status was used here to reflect short-term inpatient treatment response.

Independent variables included tumor stage (IA-IV; reference: IA), surgical grade (Level 1-4; reference: Level 1), age (continuous), total cost (continuous), out-of-pocket ratio (continuous), length of stay (continuous), chemotherapy regimen (reference: no chemotherapy), and gender (reference: female). The tumor stage variable (IA-IV) was coded as an ordinal variable ranging from 1 to 8, corresponding to stages IA, IB, IIA, IIB, IIIA, IIIB, IIIC, and IV, respectively.

Variables were selected for inclusion in the multivariable model based on clinical relevance and prior evidence regarding determinants of short-term treatment outcomes in breast cancer inpatients. Tumor stage and surgical grade represent disease severity and treatment strategy. Chemotherapy regimen reflects systemic treatment exposure. Age captures demographic variation in recovery patterns. Length of hospital stay was included as an indicator of treatment intensity and clinical complexity. Economic factors, including total cost and self-pay ratio, were incorporated to explore potential associations between healthcare utilization and short-term outcomes. Gender was included to account for possible biological or healthcare utilization differences.

There were no missing values among the variables included in the regression analysis; all records contained complete information for the selected predictors. Therefore, regression modeling was performed on a complete dataset without imputation. Continuous variables were standardized prior to inclusion in the model.

## 3 Results

### 3.1 Patient characteristics

A total of 5,052 patients hospitalized in the Department of Breast Surgery at Maternal and Child Health Hospital of Zhuhai between 2004 and 2024 were ultimately included in this study. The demographic and clinical characteristics of the patients are detailed in **Table 1** and described below.

**Table 1:** Demographic and clinical characteristics of breast cancer patients included (N=5052).

Demographic characteristics (n=5052)					Clinical characteristics (n=5052)			
Characteristics	Category	Cases (n)	Percentage (%)	Mean ± SD	Characteristics	Category	Cases (n)	Percentage (%)
Gender	Female	5028	99.5		Tumor stage	IA	1624	32.1
	Male	24	0.5			IB	1473	29.2
Age	Total	–	–	50.5 ± 10.2		IIA	760	15.0
	<40 (young group)	719	14.2	35.1 ± 3.7		IIB	519	10.3
	40-59 (peak group)	3336	66.0	49.2 ± 5.3		IIIA	299	5.9
	≥60 (elderly group)	997	19.7	65.6 ± 5.0		IIIB	208	4.1
Marital status	Married	4926	97.5			IIIC	110	2.2
	Single	74	1.5			IV	47	0.9
	Divorced	35	0.7			NA	12	0.2
	Widowed	13	0.3		Pathological type	Invasive ductal carcinoma	4485	88.8
	Not specified	4	0.1			Mucinous adenocarcinoma	104	2.1
Geographic origin	Hospital’s district	2504	49.6			Invasive lobular carcinoma	97	1.9
	Other districts within Zhuhai	1376	27.2			Ductal carcinoma in situ	77	1.5
	Other cities within Guangdong Province	808	16.0			Mammary Paget’s disease	35	0.7
	Other provinces/municipalities	331	6.6			Others*	254	5.0
	Hong Kong, Macao, or Taiwan regions	33	0.7		Clinical diagnosis (top 10)*	Upper outer quadrant breast malignancy	1669	33.0%
Ancestral origin	Guangdong	3756	74.4			Upper inner quadrant breast malignancy	751	14.9%
	Other mainland provinces	1266	25.1			Central breast malignancy	733	14.5%
	Hong Kong, Macao, or Taiwan regions	28	0.6			Upper quadrant breast malignancy	355	7.0%
	Foreign	2	<0.1			Lateral breast malignancy	298	5.9%
Occupation	Unemployed	2290	45.3			Lower inner quadrant breast malignancy	275	5.4%
	Company employees	1242	24.6			Lower outer quadrant breast malignancy	274	5.4%
	Self-employed	560	11.1			Outer quadrant breast malignancy	219	4.3%
	Retirees	288	5.7			Lower quadrant breast malignancy	166	3.3%
	Clerical and related personnel	132	2.6			Medial breast malignancy	132	2.6%
	Others	540	10.7		Comorbidities (top 10)#	Secondary malignant neoplasm of axillary lymph nodes	1114	
Payment methods	Rural-Urban Resident Basic Medical Insurance	3553	70.3			Fatty liver (not elsewhere classified)	468	
	Urban Employee Basic Medical Insurance	991	19.6			Abnormal diagnostic imaging findings of the lung	306	–
	Fully self-paying	308	6.1			Mild anemia	222	–
	Urban Resident Basic Medical Insurance	113	2.2			Drug-induced liver injury	191	–
	Commercial Medical Insurance	57	1.1			Secondary malignant neoplasm of bone	185	–
	Others	30	0.6			Secondary malignant neoplasm of multiple sites	117	–
						Secondary malignant neoplasm of the lung	100	–
						Acute posthemorrhagic anemia	86	–
						Moderate anemia	81	–

*Other pathological type and clinical diagnoses are detailed in **Appendix**.

#Comorbidities are listed by frequency of occurrence; since the same patient may have multiple comorbidities, the percentage of patients is not calculated.

#### 3.1.1 Demographic characteristics

The cohort was predominantly female (5,028 cases, 99.5%). The mean age was 50.5 ± 10.2 years. Age distribution showed that patients aged 40–59 years accounted for 66.0% (3,336 cases; mean age 49.2 ± 5.3 years), patients aged <40 years accounted for 14.2% (719 cases; mean age 35.1 ± 3.7 years), and patients aged ≥60 years accounted for 19.7% (997 cases; mean age 65.6 ± 5.0 years). Regarding marital status, 4,926 cases (97.5%) were married.

Geographic origin analysis showed that 2,504 cases (49.6%) resided in the hospital’s district, 1,376 cases (27.2%) were from other districts within Zhuhai, 808 cases (16.0%) were from other cities within Guangdong Province, 331 cases (6.6%) were from other provinces/municipalities, and 33 cases (0.7%) were from Hong Kong, Macao, or Taiwan regions. Ancestral origin distribution included 3,756 Guangdong natives (74.4%), 1,266 from other mainland provinces (25.1%), and 30 from Hong Kong/Macao/Taiwan/foreign regions (0.6%).

Occupational distribution included unemployed individuals (2,290 cases, 45.3%), company employees (1,242 cases, 24.6%), self-employed individuals (560 cases, 11.1%), retirees (288 cases, 5.7%), clerical personnel (132 cases, 2.6%), and other occupations (540 cases, 10.7%). Regarding medical payment methods, Rural-Urban Resident Basic Medical Insurance accounted for 3,553 cases (70.3%), Urban Employee Basic Medical Insurance for 991 cases (19.6%), fully self-pay for 308 cases (6.1%), and other payment methods collectively accounted for less than 4%.

#### 3.1.2 Clinical characteristics

Based on tumor stage (**Table 1**), early-stage patients (IA 1,624 cases; IB 1,473 cases; total 3,097 cases, 61.3%) and intermediate-stage patients (IIA 760 cases; IIB 519 cases; total 1,279 cases, 25.3%) constituted the majority of the cohort. Locally advanced (IIIA 299 cases; IIIB 208 cases; IIIC 110 cases) and metastatic (IV 47 cases) patients collectively accounted for 13.4%.

Pathological type distribution included invasive ductal carcinoma as the predominant type (88.8%). Mucinous adenocarcinoma accounted for 104 cases (2.1%) and invasive lobular carcinoma for 97 cases (1.9%). Non-invasive carcinomas accounted for 134 cases (2.7%), including ductal carcinoma *in situ* (77 cases) and mammary Paget’s disease (35 cases). Benign breast lesions accounted for 47 cases (1.0%), and other types accounted for 62 cases (see **Appendix**).

Tumor location was most frequently in the upper outer quadrant (1,669 cases, 33.0%), followed by upper inner quadrant (751 cases, 14.9%) and central region (733 cases, 14.5%). Detailed anatomical distribution data are provided in **Appendix**. Secondary malignant neoplasm of axillary lymph nodes was recorded in 1,114 cases. Other comorbidities included fatty liver disease (468 cases), abnormal lung imaging findings (306 cases), mild anemia (222 cases), drug-induced liver injury (191 cases), secondary malignant neoplasm of bone (185 cases), secondary malignant neoplasm of multiple sites (117 cases), secondary malignant neoplasm of the lung (100 cases), acute posthemorrhagic anemia (86 cases), and moderate anemia (81 cases).

### 3.2 Treatment patterns

Treatment patterns between 2004 and 2024 are presented in **Table 2**. Total hospitalizations increased from 49 cases in 2004 to 1,076 cases in 2024, with an average annual growth rate of 18.3% (95% CI: 15.7-21.0). In 2022, admissions increased to 948 cases compared with 187 cases in 2021.

**Table 2:** Annual trends in indicators for breast cancer treatment (2004–2024).

Year	Hospitalizations(n)	Surgeries (n)	Surgery rate (%)	Chemotherapy (n)	Chemotherapy rate (%)	Length of hospitalization (days) (Mean ± SD)
2004	49	45	91.8	35	71.4	16.9 ± 4.8
2005	52	52	100.0	39	75.0	17.9 ± 4.7
2006	49	49	100.0	40	81.6	15.5 ± 4.2
2007	54	54	100.0	26	48.1	13.8 ± 3.9
2008	60	60	100.0	50	83.3	14.3 ± 4.3
2009	84	84	100.0	57	67.9	13.1 ± 3.0
2010	103	103	100.0	42	40.8	12.9 ± 2.8
2011	89	89	100.0	36	40.4	14.1 ± 3.8
2012	62	61	98.4	28	45.2	15.2 ± 4.5
2013	114	106	93.0	17	14.9	13.0 ± 4.7
2014	137	135	98.5	32	23.4	14.4 ± 4.8
2015	134	131	97.8	20	14.9	13.7 ± 4.3
2016	149	147	98.7	29	19.5	14.9 ± 3.8
2017	132	130	98.5	62	47.0	16.2 ± 4.6
2018	143	142	99.3	110	76.9	16.1 ± 4.6
2019	137	136	99.3	108	78.8	16.9 ± 4.7
2020	158	158	100.0	100	63.3	16.0 ± 5.4
2021	187	183	97.9	124	66.3	15.7 ± 4.6
2022	948	731	77.1	522	55.1	5.7 ± 5.2
2023	1135	913	80.4	678	59.7	5.2 ± 4.9
2024	1076	844	78.4	603	56.0	4.8 ± 3.8

Surgical treatment rates ranged from 77.1% to 100% across the study period (**Table 2**).

**Figure 1:**
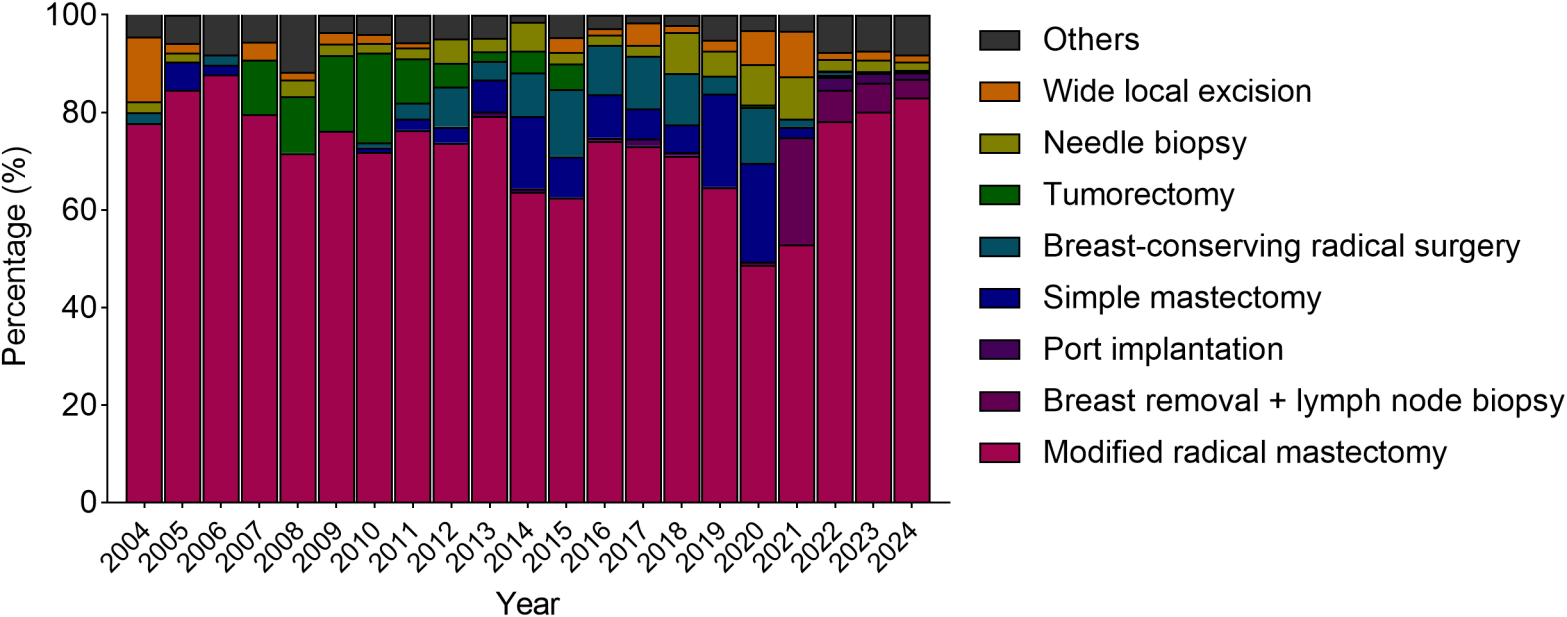
Stacked bar charts show the yearly percentage distribution of different surgical methods, including modified radical mastectomy, breast removal with lymph node biopsy, port implantation, simple mastectomy, breast-conserving radical surgery, tumorectomy, needle biopsy, wide local excision, and other procedures.

The annual distribution of surgical methods is shown in **Table 3** and **Figure 1**. From 2004-2013, modified radical mastectomy accounted for an annual average of 82.6 ± 6.3% of surgeries. radical chemotherapy accounted for 31 cumulative cases (3.1% of surgeries during that period). After 2014, the number of breast-conserving procedures increased, reaching 18 cases in 2020 (11.4% of annual surgeries). In 2024, wide local excision reached 69 cases, and port implantation accounted for 11 cases (1.3%).

**Table 3:** Annual distribution of breast cancer surgery methods (2004–2024).

Year	Modified radical mastectomy	Breast removal + lymph node biopsy	Port implantation	Simple mastectomy	Breast-conserving radical surgery	Tumorectomy	Needle biopsy	Wide local excision	Others
2004	35	0	0	0	1	0	1	6	2
2005	44	0	0	3	0	0	1	1	3
2006	43	0	0	1	1	0	0	0	4
2007	43	0	0	0	0	6	0	2	3
2008	43	0	0	0	0	7	2	1	7
2009	64	0	0	0	0	13	2	2	3
2010	74	0	0	1	1	19	2	2	4
2011	68	0	0	2	3	8	2	1	5
2012	45	0	0	2	5	3	3	0	3
2013	84	0	1	7	4	2	3	0	5
2014	86	0	1	20	12	6	8	0	2
2015	82	0	0	11	18	7	3	4	6
2016	109	1	0	13	15	0	3	2	4
2017	95	0	2	8	14	0	3	6	2
2018	101	0	1	8	15	0	12	2	3
2019	88	0	0	26	5	0	7	3	7
2020	77	1	0	32	18	1	13	11	5
2021	97	40	0	4	3	0	16	17	6
2022	572	47	19	3	5	1	18	10	56
2023	732	54	18	1	1	1	22	17	67
2024	701	32	11	2	1	2	14	12	69

Annual chemotherapy distribution is shown in **Table 4**. Adjuvant chemotherapy accounted for 69.3% of total chemotherapy. Radical chemotherapy predominated during 2004-2017 (63.2 ± 22.1%). Neoadjuvant chemotherapy increased after 2018, reaching 32 cases in 2024 (5.3% of annual chemotherapy).

**Table 4:** Annual distribution of chemotherapy methods (2004–2024).

Year	Adjuvant	Radical	Palliative	Neoadjuvant	Year	Adjuvant	Radical	Palliative	Neoadjuvant
2004	2	31	0	2	2014	20	9	1	2
2005	30	8	1	0	2015	10	7	2	1
2006	34	6	0	0	2016	16	8	2	3
2007	17	9	0	0	2017	37	18	2	5
2008	49	0	1	0	2018	79	18	2	11
2009	50	3	0	4	2019	78	11	4	15
2010	28	12	0	2	2020	77	5	2	16
2011	12	24	0	0	2021	90	11	4	19
2012	18	8	1	1	2022	456	25	16	25
2013	11	4	1	1	2023	586	35	22	35
					2024	529	28	14	32

**Figure 2:**
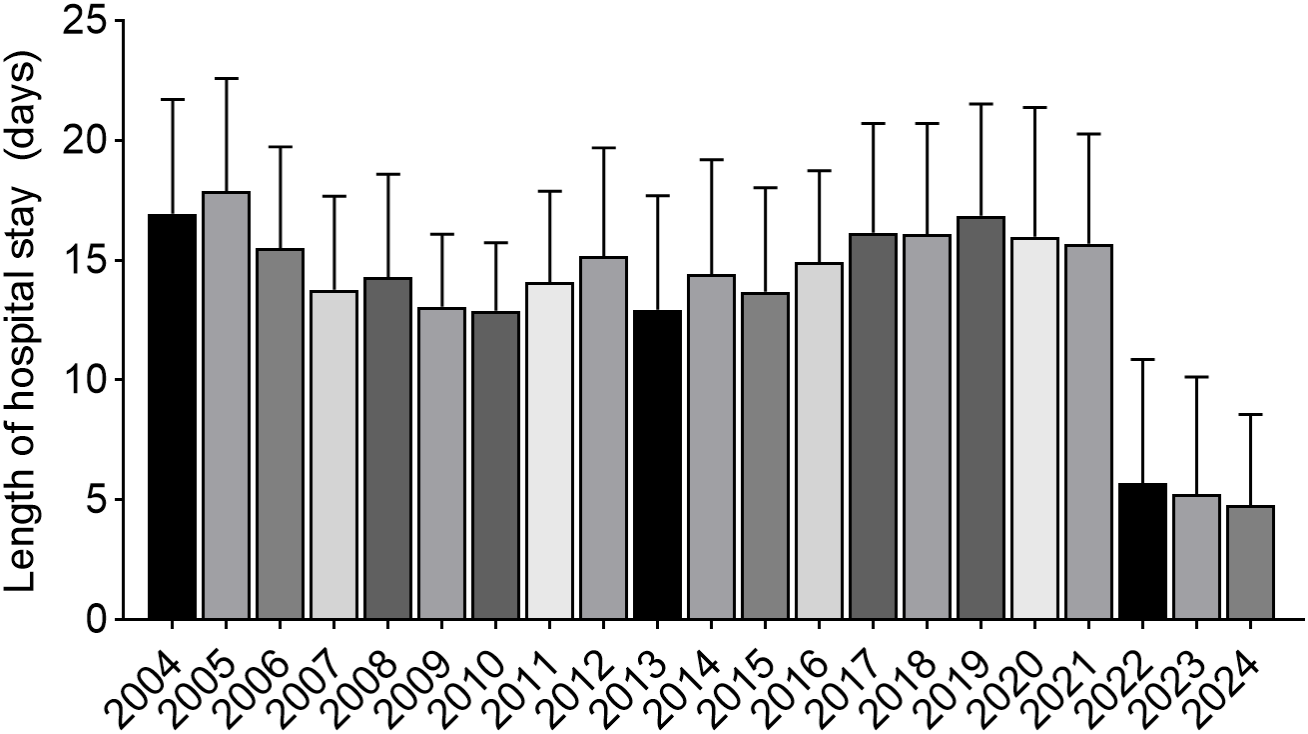
Bars represent the mean length of hospital stay (days) for each year. Error bars indicate standard deviation.

Average length of stay decreased from 16.9 ± 4.8 days in 2004 to 4.8 ± 3.8 days in 2024, representing a 71.6% reduction (p < 0.001) (**Figure 2**). The standard deviation of hospitalization duration decreased from 4.7 ± 0.7 days in earlier years to 3.8 days in 2024. .

### 3.3 Evolution of medical cost and self-pay ratio (2004–2024)

**Figure 3:**
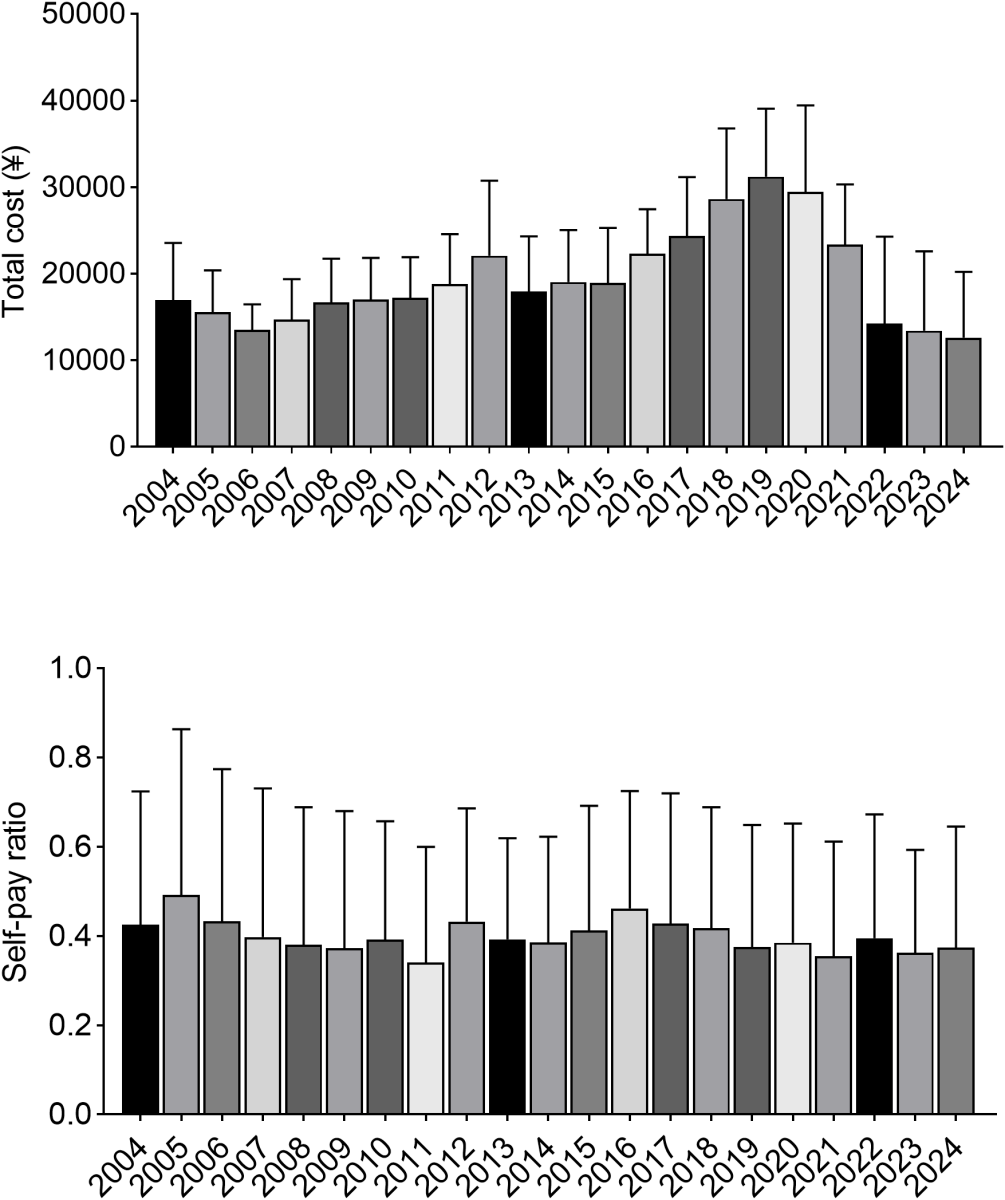
The upper panel shows the mean total hospitalization cost (¥) by year, and the lower panel shows the mean self-pay ratio by year. Error bars indicate standard deviation.

The annual evolution of medical expense structure, total cost, and self-pay ratio is shown in **Table 5** and **Figure 3**. Total cost increased from ¥16,981 in 2004 to a peak of ¥31,201 in 2019, and then declined to ¥12,809-¥14,211 during 2022-2024.

**Table 5:** Evolution of medical expense structure (2004–2024).

Year	Average total cost	Standard deviation of total cost	Average self-pay ratio	Standard deviation of self-pay ratio	Diagnostic costs (ratio)	Treatment costs (ratio)	Medication costs (ratio)	Nursing costs (ratio)	Other (ratio)
2004	16981.42	6565.89	42.6%	29.8%	18.8%	52.2%	23.6%	4.9%	0.5%
2005	15564.00	4808.92	49.2%	37.1%	16.4%	51.1%	26.3%	6.0%	0.3%
2006	13523.87	2952.00	43.3%	34.0%	25.1%	45.5%	24.1%	5.1%	0.3%
2007	14713.43	4686.38	39.8%	33.3%	24.0%	25.9%	45.4%	4.7%	0.0%
2008	16663.14	5064.47	38.1%	30.7%	22.6%	29.0%	43.4%	4.9%	0.1%
2009	17011.38	4836.75	37.2%	30.8%	22.8%	27.8%	44.1%	5.0%	0.3%
2010	17241.31	4689.74	39.2%	26.5%	19.2%	20.1%	56.1%	4.4%	0.2%
2011	18799.95	5761.36	34.1%	25.8%	19.8%	19.6%	56.0%	4.4%	0.2%
2012	22065.39	8677.16	43.3%	25.3%	23.3%	23.7%	48.0%	4.6%	0.3%
2013	17940.20	6364.15	39.3%	22.6%	22.6%	26.2%	46.5%	4.5%	0.2%
2014	19041.26	5993.85	38.6%	23.7%	19.7%	22.9%	52.7%	4.5%	0.2%
2015	18948.93	6357.26	41.3%	27.9%	23.4%	25.0%	46.7%	4.6%	0.3%
2016	22282.53	5164.80	46.2%	26.3%	18.9%	19.3%	56.6%	5.0%	0.2%
2017	24357.89	6804.91	42.9%	29.1%	22.9%	22.4%	49.8%	4.5%	0.3%
2018	28632.35	8148.53	41.7%	27.1%	20.3%	23.1%	51.3%	5.0%	0.2%
2019	31200.96	7885.64	37.6%	27.3%	21.4%	18.9%	54.8%	4.8%	0.2%
2020	29447.50	10018.06	38.5%	26.7%	27.1%	21.1%	46.7%	4.9%	0.2%
2021	23348.90	6976.42	35.5%	25.7%	22.2%	20.1%	52.9%	4.5%	0.2%
2022	14211.71	10050.33	39.5%	27.7%	17.7%	14.2%	63.5%	4.4%	0.2%
2023	13650.55	9244.08	36.2%	23.1%	17.8%	51.7%	23.1%	6.6%	0.7%
2024	12808.94	7648.52	37.4%	27.2%	19.6%	51.9%	22.9%	5.3%	0.3%

During 2004-2013, treatment costs (surgery/radiotherapy) accounted for an annual average of 45.1% ± 8.7%, while medication costs accounted for 35.8% ± 11.9%. In 2022, medication costs accounted for 63.5% of total cost. In 2023, treatment costs accounted for 51.7%. Surgical volume in 2023 was 913 cases, and local extended resection accounted for 8.2%.

The out-of-pocket ratio decreased from 49.2% ± 37.1% in 2005 to 37.4% ± 27.2% in 2024. The standard deviation of out-of-pocket proportion decreased from 0.30 to 0.23. During 2020-2024, out-of-pocket proportions remained between 37% and 39%.

### 3.4 Logistic regression analysis of factors influencing treatment efficacy

Multivariable logistic regression was performed to identify factors associated with treatment efficacy (**Table 6**). Model fit statistics were: AIC = 1089.9; χ² = 136.5, p < 0.001.

**Table 6:** Results of logistic regression analysis.

Variable	Estimate	Std. Error	z value	Pr(>|z|)	Significance	95% CI (2.50%)	95% CI (97.50%)	OR
(Intercept)	3.152	0.5629	5.6	<0.001	***	7.7581	70.4699	23.3818
Stage2	0.058	0.2388	0.243	0.808		0.6636	1.6924	1.0598
Stage3	-0.1392	0.2792	-0.498	0.6182		0.5034	1.5039	0.8701
Stage4	0.784	0.4337	1.808	0.0707	.	0.936	5.125	2.1902
Stage5	-0.1114	0.3911	-0.285	0.7758		0.4156	1.9255	0.8946
Stage6	-0.2293	0.4408	-0.52	0.6029		0.3351	1.8863	0.7951
Stage7	-0.7293	0.4929	-1.479	0.139		0.1835	1.2673	0.4823
Stage8	13.8	560.5	0.025	0.9804		/	/	/
Surgical grade2	-0.0014	0.2097	-0.007	0.9946		0.6621	1.5061	0.9986
Surgical grade3	0.6479	0.2737	2.367	0.0179	*	1.1179	3.2683	1.9115
Surgical grade4	-4.834	0.7021	-6.885	<0.001	***	0.002	0.0315	0.008
Age	0.0008	0.0093	0.085	0.932		0.9827	1.0192	1.0008
Total cost	0	0	1.039	0.2987		0.99999	1.00004	1
Self pay ratio	-0.0084	0.3405	-0.025	0.9802		0.5088	1.9327	0.9916
Gender Male	-1.028	0.7746	-1.327	0.1845		0.0784	1.6326	0.3577
Length of hospital stay	-0.0357	0.0141	-2.529	0.0114	*	0.9386	0.992	0.9649
Chemotherapy method Adjuvant	1.771	0.3196	5.541	<0.001	***	3.1403	10.9899	5.8747
Chemotherapy method Radical	1.645	1.015	1.622	0.1049		0.7095	37.8572	5.1827
Chemotherapy method Palliative	1.058	0.5941	1.781	0.0749	.	0.8993	9.2311	2.8813
Chemotherapy method Neoadjuvant	0.477	0.3256	1.465	0.1429		0.8512	3.05	1.6112

p < 0.1; * p < 0.05; ** p < 0.01; *** p < 0.001.

Tumor stage showed no statistically significant association with outcomes. Stage IIB showed OR = 2.19 (p = 0.071). Stage IV was excluded due to insufficient sample size.

Compared with Grade 1 surgery, Grade 4 (palliative resection) showed OR = 0.008 (95% CI: 0.002-0.031, p < 0.001), and Grade 3 surgery showed OR = 1.91 (95% CI: 1.12-3.27, p = 0.018).

Among chemotherapy strategies, adjuvant chemotherapy showed OR = 5.87 (95% CI: 3.14-10.99, p < 0.001 vs. no chemotherapy). Length of stay showed OR = 0.96 (95% CI: 0.94-0.99, p = 0.011). Age, gender, total costs, and self-pay ratio were not statistically significant.

## 4 Discussion

Current studies indicate that the peak age of breast cancer incidence in China is between 40–59 years (11, 12), which is consistent with our findings. In contrast, the high-risk age group in the United States is over 70 years (13); in Belgium, incidence increases after 50 and becomes more concentrated beyond 70 (14); and in Turkey, the majority of cases (40.7%) occur in women aged 51-70 (15). While breast cancer in Western countries predominantly occurs after menopause, in China patients are largely perimenopausal, which may be related to national trends of delayed marriage, later childbearing, and declining fertility rates (12).

Anatomically, the upper-outer quadrant contains the highest proportion of glandular tissue and is most susceptible to malignancy (16), consistent with the distribution observed in our data. Invasive ductal carcinoma (IDC) accounts for 60–75% of cases globally, whereas invasive lobular carcinoma (ILC) accounts for 10-15% (17, 18). Our cohort showed a predominance of IDC (88.8%) and a lower proportion of ILC (1.9%), suggesting possible regional variations.

A key finding was the observed transformation in treatment patterns: the rate of breast-conserving surgery increased substantially from 3.1% (2004-2013) to 21.7% (2024). This shift co-occurred with two changes: first, the rapid adoption of neoadjuvant chemotherapy (38.7% average annual growth since 2018), which may have expanded surgical options for the 20.5% of Stage III patients; second, the promotion of day-care models, which was associated with a 71.6% reduction in median length of stay (p<0.001) and accompanied by higher surgical volume. These changes may reflect progress toward addressing the historically low rates of breast conservation in China. Nonetheless, mastectomy remains the predominant approach nationally, accounting for 88.8% of primary breast cancer surgeries—a proportion substantially higher than that reported in the U.S. (36%) (19). Even in more developed regions such as Beijing and Shanghai, adoption rates of breast-conserving surgery remained as low as 24.3% (20). Limited access to radiotherapy resources, particularly in rural areas, may partly explain the slower uptake in China (21). In addition, the low recorded rate of core needle biopsy in our inpatient data likely reflects its performance in outpatient settings.

We also observed substantial changes in hospitalization efficiency and cost structure. Total medical costs declined after 2019, coinciding with shorter hospital stays. Medication costs accounted for a higher proportion of total expenditure in 2022, possibly reflecting broader adoption of targeted therapies, whereas treatment-related costs increased again in 2023. The out-of-pocket ratio decreased gradually over time but remained relatively stable after 2020. These findings highlight structural shifts in healthcare expenditure rather than simple reductions in overall cost. The reported average cost per new breast cancer patient in China is $1,216 (≈¥8,369), and $2,835 (≈¥19,513) in coastal cities (12), consistent with our observed average cost of ¥18,569.7.

A sharp increase in hospitalizations was observed in 2022. This occurred within the broader context of rising healthcare utilization in China, where national inpatient admissions reached approximately 301.9 million in 2023 and the population hospitalization rate exceeded 20% (22). Expanded medical insurance coverage and adjustments in healthcare utilization following pandemic-related policy changes may have influenced inpatient demand (23). However, the specific drivers of the 2022 surge in breast cancer admissions cannot be determined from our data and should be interpreted cautiously.

Chemotherapy strategies also evolved over time. Neoadjuvant chemotherapy increased after 2018 and aligns with contemporary CSCO guideline recommendations for locally advanced disease (20). Adjuvant chemotherapy remains common in China, with 81.4% of invasive breast cancer patients reportedly receiving it (24), although discontinuation rates remain notable (24). Drug reimbursement policies heavily influence treatment choices, and high costs may explain why nearly half of the patients in our results did not undergo chemotherapy. Palliative care is underutilized in China, potentially due to cultural taboos around discussing death (12), which is also reflected in our data. Neoadjuvant therapy refers to systemic treatment (e.g., chemotherapy, endocrine therapy, targeted therapy) administered before primary local treatment such as surgery (25). It is generally indicated only for patients with locally advanced disease (25), which may explain the relatively low number of patients receiving neoadjuvant chemotherapy in our study.

In multivariable analysis, adjuvant chemotherapy was associated with higher odds of favorable short-term in-hospital outcome, whereas palliative resection was associated with unfavorable outcomes. Longer length of stay was associated with lower odds of favorable outcome. These findings reflect associations rather than causal effects and should be interpreted within the context of short-term discharge status.

This study provides a comprehensive longitudinal overview of inpatient breast cancer management in a rapidly developing coastal region. Zhuhai’s experience with expanding insurance coverage and reduced hospitalization duration may offer reference value for similar settings. Several limitations should be acknowledged. First, this was a single-center study based exclusively on hospitalized patients. Patients managed entirely in outpatient settings were not captured; therefore, the reported treatment patterns and resource utilization may not fully represent the broader breast cancer population, particularly those with early-stage disease. In addition, as a regional referral center, our hospital may receive a higher proportion of complex or referred cases, and potential referral bias cannot be excluded. Second, long-term endpoints such as recurrence and survival were unavailable. Treatment outcome was defined based on discharge status, categorized in routine clinical practice as “cured,” “improved,” “not improved,” or “death,” and dichotomized for analysis. Although long-term survival is emphasized in clinical guidelines and quality indicators (26, 27), discharge status serves as a pragmatic indicator of short-term inpatient treatment response. Finally, molecular subtype data (e.g., ER, PR, HER2 status) were unavailable due to equipment constraints. Given that molecular characteristics strongly influence therapeutic decision-making, the absence of these data may limit interpretation of treatment patterns across biologically distinct subgroups. Future multi-center studies incorporating molecular profiles and long-term follow-up would further enhance generalizability and strengthen outcome assessment.

## 5 Conclusion

This 20-year analysis outlines evolving inpatient breast cancer management in a rapidly developing coastal region of China. Shifts in surgical practice, chemotherapy use, and hospitalization efficiency reflect adaptation to clinical guidelines and healthcare reform. These findings have implications for optimizing resource allocation and care delivery. Future multi-center prospective studies incorporating molecular subtypes and long-term outcomes are warranted.

## Data Availability

The raw data supporting the conclusions of this article will be made available by the authors, without undue reservation.
