# Correction: Retrospective study of hospitalized breast cancer patients in a Zhuhai-based hospital: analysis spanning over 20 years

**DOI:** 10.3389/fonc.2026.1839941

**Published:** 2026-04-09

**Authors:** Ruixin Fan, Yu Zheng, Xiangfeng Zhao, Juan Lin, Xueping Lu, Pan Hong, Weirong Chen

**Affiliations:** 1 Breast Surgery Department, Zhuhai Center for Maternal and Child Health Care (Zhuhai Women and Children’s Hospital), Zhuhai, Guangdong, China; 2 Department of Breast and Thyroid Surgery, Union Hospital, Tongji Medical College, Huazhong University of Science and Technology, Wuhan, China; 3 Department of Orthopaedic Surgery, Union Hospital, Tongji Medical College, Huazhong University of Science and Technology, Wuhan, China

**Keywords:** breast cancer, epidemiology, healthcare costs, retrospective study, treatment patterns

Affiliation “Breast Surgery Department, Zhuhai Center for Maternal and Child Health Care (Zhuhai Women and Children’s Hospital), Zhuhai, Guangdong, China” was erroneously given as “Department of Breast Surgery, Maternal and Child Health Hospital of Zhuhai, Zhuhai, China”.

The original version of this article has been updated.

